# Clinical Effects of Xihuang Pill Combined with Chemotherapy in Patients with Advanced Colorectal Cancer

**DOI:** 10.1155/2017/5936086

**Published:** 2017-03-28

**Authors:** Dan Yu, Guang Yu An

**Affiliations:** Department of Oncology, Beijing Chaoyang Hospital, Capital Medical University, Beijing, China

## Abstract

*Objective*. To investigate the therapeutic effects of Xihuang pill combined with chemotherapy on advanced colorectal cancer.* Methods.* Sixty-three patients with advanced colorectal cancer were divided into an experimental group (*n* = 32) and control group (*n* = 31). Patients in the experimental group were treated with traditional Chinese medicine combined with Western medicine (i.e., Xihuang pill with FOLFOX or FOLFIRI chemotherapy), and those in the control group were treated with FOLFOX or FOLFIRI chemotherapy alone. Changes in therapeutic efficacy, side effects, blood coagulation function, and quality of life (QOL) were compared between the two groups.* Results*. The response rate was higher in the experimental than control group (*P* = 0.011). The QOL score in the experimental group was significantly lower after treatment than before treatment (*P* = 0.003), while no significant change was found in the control group. In the experimental group, the posttreatment activated partial thromboplastin time and prothrombin time after treatment were 30.05 ± 3.85 and 10.40 ± 1.25 s, respectively, which were prolonged compared with those before treatment (29.12 ± 4.03 and 9.85 ± 1.00 s; *P* = 0.010 and 0.021, respectively).* Conclusion*. In patients with advanced colorectal cancer, Xihuang pill combined with chemotherapy can significantly enhance the therapeutic effects compared with chemotherapy alone and improve patients' QOL and hypercoagulability.

## 1. Introduction

Colorectal cancer (CRC) is one of the most common tumors worldwide. According to epidemiological statistics, both the incidence and mortality rate of CRC ranked third among all malignant tumors in the United States in 2015 [1]. CRC is diagnosed at an advanced stage in most patients because of its occult symptoms, and the best opportunity for surgical treatment is thus lost. Although FOLFOX and FOFIRI chemotherapy are the international standards for the treatment of advanced CRC, the effect of chemotherapy is poor in most of these patients because of the high tumor load and low sensitivity to treatment as well as the patients' poor quality of life (QOL), intolerance to high-intensity chemotherapy, and hypercoagulable state with resultant predisposition to thromboembolic disease. Therefore, an understanding of the antitumor effects of traditional Chinese medicine is of great clinical significance. Xihuang pill, a classic anticancer Chinese medicine, contains four rare Chinese herbs: bezoar, musk, frankincense, and myrrh. This study was designed to investigate the effects of Xihuang pill combined with chemotherapy on the treatment efficacy, QOL improvement, and incidence of acute thrombosis in patients with advanced CRC.

## 2. Materials and Methods

### 2.1. Clinical Data

This study included 63 patients (34 male, 29 female) with advanced metastatic CRC admitted to the Oncology Department of Beijing Chaoyang Hospital from January 2013 to January 2016. The numbers of patients with pulmonary metastasis, liver metastasis, abdominal cavity and retroperitoneal lymph node metastasis, and peritoneal implantation metastasis were 24, 37, 41, and 28, respectively. The patients' ages ranged from 29 to 72 years (Table 1). All patients' diagnoses were confirmed by pathological examination, and none had a history of other tumors.

### 2.2. Inclusion and Exclusion Criteria

 The inclusion criteria were as follows:

- Pathological diagnosis of CRC.
- Treatment-naïvety with no history of radiotherapy, chemotherapy, or any type of antitumor therapy (for patients who developed relapse after adjuvant chemotherapy following radical resection, the last chemotherapy was completed within 6 months).
- Eastern Cooperative Oncology Group (ECOG) physical status of ≤2 (Table 2).
- Expected survival time of ≥3 months.
- Age of 20 to 79 years.
- Measurable lesions (ability to measure at least one diameter line) identifiable by imaging or physical examination; maximum lesion diameter of ≥2 cm under conventional detection conditions or ≥1 cm by computed tomography (CT).
- Neutrophilic granulocyte count of ≥1.5 × 10^9^/L, platelet count of 80 × 10^9^/L, hemoglobin concentration of ≥80 g/L, serum bilirubin concentration of ≤1.5 times the upper limit of normal, and alanine aminotransferase/aspartate aminotransferase concentrations of ≤2.5 times the upper limit of normal (≤5.0 times for patients with liver metastasis).

The exclusion criteria were as follows:

- Under treatment with other chemotherapies or radiotherapies.
- Pregnancy, lactation.
- Severe liver and renal impairment.
- History of an uncontrollable mental disorder.
- Severe acute cardiac and cerebral vascular diseases.

### 2.3. Experimental Methods

Using a completely random grouping method, the patients were divided into an experimental group (*n* = 32) and a control group (*n* = 31). There were no significant differences in sex, age, or ECOG physical status between the two groups. Patients in the control group were treated with chemotherapy alone using either FOLFOX (LOHP at 85 mg/m^2^ on day 1 + CF at 200 mg/m^2^ on days 1 and 2 + 5-FU at 400 mg/m^2^ on days 1 and 2 + 5-FU at 1200 mg/m^2^ in a continuous intravenous infusion for 44 h every 14 days) or FOLFIRI (CPT-11 at 180 mg/m^2^ on day 1 + CF at 200 mg/m^2^ on days 1 and 2 + 5-FU at 400 mg/m^2^ on days 1 and 2 + 5-FU at 1200 mg/m^2^ in a continuous intravenous infusion for 44 h every 14 days). Patients in the experimental group were treated with one of the above chemotherapy regimens plus oral Xihuang pill (3 g/bottle; Beijing Tongrentang Group Co., Ltd.) administered at 3 g twice a day. In both groups, treatment efficacy was determined after four cycles of chemotherapy (one course of treatment was 56 days).

### 2.4. Clinical Outcome Measures

 The clinical outcome measures were as follows:

- Routine blood tests, biochemical tests for liver and kidney function, and blood coagulation function testing before and after four cycles of chemotherapy.
- Imaging examinations, such as CT and magnetic resonance imaging, before and after four cycles of chemotherapy.
- Evaluation of ECOG physical status and chemotherapy-related toxicity, such as bone marrow suppression and gastrointestinal reactions. Toxicity evaluation was performed with reference to the Common Terminology Criteria for Adverse Events.

### 2.5. Response Evaluation Criteria in Solid Tumors (RECIST) Evaluation Criteria (Table 3)

The following RECIST criteria were assessed:

- Measurable lesions (presence of at least one lesion with a diameter that could be accurately measured; the longest diameter was measured)
- Tumorous Lesions
  - Longest diameter of ≥10 mm by vernier caliper during clinical examination
  - Longest diameter of ≥20 mm on chest radiograph and ≥10 mm on spiral CT (thinner scan is used if ≤5 mm on spiral CT)
- Malignant Lymph Nodes
  - Shortest lymph node diameter of ≥15 mm on spiral CT (thinner scan is used if ≤5 mm on spiral CT)
- Target lesion selection
- Selection of up to five measurable lesions, with up to two for each organ
- Target lesion evaluation

### 2.6. Statistical Methods

Statistical analysis was performed using SPSS 13.0 software (SPSS Inc., Chicago, IL, USA). Measurement data are presented as mean ± standard deviation and enumeration data as frequency (rate). An independent-sample *t*-test was used for between-group comparisons, and a paired *t*-test was used for pre- and posttreatment intragroup comparisons. The data were not consistent with normality, and comparisons between groups were performed with the Mann–Whitney *U* test. The paired data were compared with the Wilcoxon signed rank test. A *P* value of <0.05 was considered statistically significant.

## 3. Results

### 3.1. Therapeutic Efficacy

The response rate in the experimental group (*n* = 32) was 46.88% (complete response, *n* = 0; partial response, *n* = 15; stable disease, *n* = 19; progressive disease, *n* = 8). The response rate in the control group (*n* = 31) was 22.58% (complete response, *n* = 0; partial response, *n* = 7; stable disease, *n* = 13; progressive disease, *n* = 11). The difference in the response rates between the two groups was statistically significant (Table 4).

### 3.2. Tumor Markers

Before treatment, the carcinoembryonic antigen (CEA) concentration in the experimental and control groups was 66.5 and 68.0 ng/ml, respectively, with no significant difference. The CEA concentration was lower after treatment than before treatment in both the experimental and control groups (25.0 and 59.0 ng/ml, respectively); however, the difference was only statistically significant in the experimental group (Table 5).

### 3.3. Side Effects

No significant differences in side effects, including bone marrow suppression, gastrointestinal reactions, and abnormal liver and renal function, were found between the two groups (*P* > 0.05) (Table 6).

### 3.4. Coagulation Function

There were no statistically significant differences in the pretreatment activated partial thromboplastin time (APTT), prothrombin time (PT), or D-dimer concentration between the two groups (Table 7). In the experimental group, the APTT and PT were longer after treatment than before treatment, while the D-dimer concentration was lower. These differences were statistically significant. In the control group, however, no significant changes were observed in the APTT, PT, or D-dimer concentration before and after treatment (Table 8).

### 3.5. QOL

The pretreatment ECOG physical status in the control and experimental groups was 1.06 ± 0.77 and 1.09 ± 0.73, respectively (*P* = 0.878), without statistical significance. That after treatment was 1.16 ± 0.93 in the control group and 0.75 ± 0.57 in the experimental group. The ECOG physical status in the experimental group was significantly lower after treatment than before treatment, while in the control group, there was no significant change in the ECOG physical status before and after treatment (Table 9).

## 4. Discussion

Xihuang pill, a classic anticancer Chinese medicine, is composed of four rare Chinese herbs: bezoar, musk, frankincense, and myrrh. The combination of these four drugs powerfully clears away heat and toxic material and promotes blood circulation to remove blood stasis. These effects are made possible by the ability of bezoar to clear heat and detoxify, the ability of musk to activate blood stagnation and expel blood stasis, and the ability of frankincense and myrrh to promote blood and vital energy circulation and decrease swelling and pain. The four drugs are combined and made into pills with steamed rhubarb rice, which protects the physiological function of the stomach and eliminates pathogenic factors without injury. Xihuang pill alone or combined with other Western antitumor therapies is applied in the treatment of various malignant tumors and shows preferable antitumor effects on breast cancer, lymphoma, esophageal cancer, ovarian cancer, and primary liver cancer [2–7].

Few clinical studies on the antitumor effects of Xihuang pill in the treatment of CRC have been reported to date. The present study has shown that Xihuang pill combined with chemotherapy (experimental group) has a significantly better response rate than chemotherapy alone (control group) when used as first-line treatment for advanced CRC, indicating that Xihuang pill is a promising adjuvant therapy that enhances the effectiveness of antitumor treatment. The concentration of CEA, a sensitive tumor marker of CRC, decreased in both groups after treatment; however, while the CEA concentration in the experimental group was significantly lower than that before treatment, there was no significant change in the control group. This further proves that Xihuang pill combined with chemotherapy provides better treatment control and reduces the tumor load further than does chemotherapy alone. Several possible reasons for this increased efficacy are as follows. First, Xihuang pill is a compounded Chinese medicine preparation that consists of four herbs: bezoar, musk, frankincense, and myrrh. Frankincense and myrrh contain a substantial amount of volatile oils, mainly a variety of alkenes, including *β*-, *γ*-, and *δ*-elemene. Alkenes exert an antitumor effect through their cytotoxic effects and enhance the immune function of the body [8, 9]. Detailed investigations of the antitumor mechanism of Xihuang pill have been performed in recent years. Duo et al. [10] established a subcutaneously transplanted human colon cancer model in nude mice and found that Xihuang pill inhibited the phosphorylation of extracellular signal-regulated protein kinases 1/2 (ERK1/2) and repressed the mitogen activated protein kinase signaling pathway, thereby inhibiting the proliferation of the human colon cancer xenografts and delaying tumor growth. These results demonstrate that Xihuang pill exerts an inhibitory effect on tumor cell proliferation. In addition, Xihuang pill regulates the expression of E and N cadherin through regulation of the ERK pathway and ZEB1-SCRIB cycle and represses the epithelial-mesenchymal transition of human colon cancer cells, thus inhibiting the invasion and metastasis of tumor cells [11].

In the present study, the ECOG performance status in the experimental group was significantly higher after treatment than before treatment, and the difference was statistically significant. This suggests that Xihuang pill combined with chemotherapy can significantly improve patients' QOL and increase the survival benefit. The main mechanism may involve the potent antitumor effect of Xihuang pill. Patients' QOL may be improved when their tumors are under control and symptoms are relieved. Additionally, Xihuang pill may also improve QOL of patients with advanced cancer by enhancing the body's immune function. Our study findings demonstrate that Xihuang pill plays a regulatory role in the tumor mass and immune microenvironment. In a previous study, ethanol extract of Xihuang pill increased the expression of interleukin-2 and interferon-*γ*, decreased the expression of interleukin-10, and regulated the ratios of CD3+, CD4+, and CD8+ T lymphocytes in the peripheral blood of rats with tumors [12]. Lan and Peng [13] included Xihuang pill combined with other antitumor therapy in the treatment of postoperative patients with CRC and patients with nasopharyngeal carcinoma undergoing chemotherapy, with an average observation time of 2 years and 7 months. The results showed that long-term use of Xihuang pill after conventional antitumor therapies helps to improve symptoms and prevent the recurrence and metastasis of tumors.

Approximately 90% of patients with malignant tumors are reportedly in a state of hypercoagulability. The application of traditional Chinese medicine that promotes blood circulation to remove blood stasis may improve this hypercoagulability and relieve or prevent its associated adverse events, therefore enhancing patients' QOL. In this study, the effect of Xihuang pill on blood coagulation function was also explored; in the experimental group, the APTT and PT were higher and the D-dimer concentration was lower after treatment than before treatment. The differences were of statistical significance. Therefore, this study has demonstrated that Xihuang pill has a significant curative effect on the hypercoagulative state of patients with CRC. Liu et al. [14] evaluated a subgroup of 80 patients with malignant tumors and found that application of Xihuang pill could safely improve the blood hypercoagulative state and reduce the platelet count and aggregation rate. According to the basic treatment principles of malignant tumors documented in traditional Chinese medicine literature (i.e., reducing phlegm and resolving masses, promoting blood circulation and detoxification, promoting the body's resistance, eliminating pathogenic factors, and purging and tonifying in combination [15]), Xihuang pill can improve patients' hypercoagulative state by its therapeutic effects of heat-clearing and detoxification, activating blood circulation and removing blood stasis, decreasing swelling, and relieving pain.

In summary, the results of this study show that Xihuang pill may improve the response rate, inhibit tumor growth, enhance the QOL, and reverse the hypercoagulative state in patients with CRC.

## Figures and Tables

**Table 1:** General situation.

	Experimental group	Control group	*P*
Gender			0.712
Male	18 (52.9)	16 (47.1)	
Female	14 (48.3)	15 (51.7)	
Age (year)	58.13 ± 10.50	58.45 ± 10.01	0.900
ECOG	1.09 ± 0.73	1.06 ± 0.77	0.878

**Table 2:** Performance status by ECOG score standard (ZPS, 5 points).

Grade	Performance status
0	Fully active, able to carry on all predisease performance without restriction
1	Restricted in physically strenuous activity but ambulatory and able to carry out work of a light or sedentary nature, for example, light house work, office work
2	Ambulatory and capable of all self-care but unable to carry out any work activities, up to and about more than 50% of waking hours
3	Capable of only limited self-care; confined to bed or chair more than 50% of waking hours
4	Cannot carry on any self-care, totally confined to bed or chair
5	Dead

**Table 3:** Evaluation criteria of chemotherapy for solid tumors.

Response assessment	Evaluation criteria
CR	All target lesions have disappeared during the course of treatment and the pathological lymph nodes are reduced to <10 mm
PR	Decreases of at least 30% from base line have been noted in the sum of LD of target lesions
PD	There has been an increase of at least 20% in the sum of the LD of targeted lesions, and it is emphasized that the absolute value of the increased sum of LD is 5 mm, or new lesions appeared
SD	Between PD and PR

**Table 4:** Comparison of response rates.

Group	PR	SD	PD	RR (%)	*P* value
Experimental group	15	9	8	46.88	0.011
Control group	7	13	11	22.58	

**Table 5:** Comparison of CEA.

Groups	*n*	Pretreatment; ng/ml	Posttreatment; ng/ml	*P*
Experimental group	32	66.5 (27.3, 462.5)	25.0 (15.5, 117.5)	0.000
Control group	31	68.0 (25.0, 523.0)	59.0 (32.0, 410.0)	0.074
*P*		0.837	0.033	

**Table 6:** Side effects (*n* = 63).

Group	Bone marrow suppression	Gastrointestinal reaction	Abnormal liver function	Abnormal renal function
Experimental group	20	28	8	0
Control group	19	29	8	0
*P* value	0.674	1.000	0.941	—

**Table 7:** Coagulation function before treatment (*n* = 63).

Group	APTT (seconds)	PT (seconds)	D-dimer (mg/L)
Experimental group	29.12 ± 4.03	9.85 ± 1.00	1.26 ± 0.83
Control group	28.86 ± 3.93	9.61 ± 0.95	1.42 ± 1.39
*P* value	0.798	0.340	0.578

**Table 8:** Changes of coagulation function before and after treatment (*n* = 63).

Group		APTT (seconds)	PT (seconds)	D-dimer (mg/L)
Experimental group	Before treatment	29.12 ± 4.03	9.85 ± 1.00	1.26 ± 0.83
	After treatment	30.05 ± 3.85	10.40 ± 1.25	0.85 ± 0.44
	*P* value	0.010	0.021	0.010
Control group	Before treatment	28.86 ± 3.93	9.61 ± 0.95	1.42 ± 1.39
	After treatment	29.09 ± 3.71	9.75 ± 0.92	1.37 ± 1.20
	*P* value	0.053	0.155	0.625

**Table 9:** Changes of ECOG (*n* = 63).

Group	Time	ECOG	*P* value
Experimental group	Before treatment	1.09 ± 0.73	
	After treatment	0.75 ± 0.57	0.003
Control group	Before treatment	1.06 ± 0.77	
	After treatment	1.16 ± 0.93	0.5
